# The Influence of Beta-2 Adrenergic Receptor Gene Polymorphisms on Albuterol Therapy for Patients With Asthma: Protocol for a Systematic Review and Meta-Analysis

**DOI:** 10.2196/14759

**Published:** 2019-09-16

**Authors:** Keiko Hikino, Shinobu Kobayashi, Erika Ota, Taisei Mushiroda, Tohru Kobayashi

**Affiliations:** 1 Laboratory for Pharmacogenomics, RIKEN Center for Integrative Medical Sciences Kanagawa Japan; 2 Department of Social Medicine, National Center for Child Health and Development Tokyo Japan; 3 Global Health Nursing Graduate School of Nursing Sciences St. Luke's International University Tokyo Japan; 4 Department of Management and Strategy Center for Clinical Research and Development National Center for Child Health and Development Tokyo Japan

**Keywords:** pharmacogenomics, polymorphisms, ADRB2, albuterol, asthma, response

## Abstract

**Background:**

Albuterol is one of the most frequently used medications in clinical practice and seeing varying responses to albuterol between individuals is not uncommon. Multiple studies have been conducted to investigate the associations of differing responses due to albuterol, particularly with regards to the two nonsynonymous single nucleotide polymorphisms (SNPs) at positions 16 (Arg16Gly: substitution of arginine to glycine at position 16; rs1042713) and 27 (Glu27Gln: substitution of glutamic acid to glutamine at position 27; rs1042714) on the β-2 adrenergic receptor (ADRB2) gene. However, the directions of the correlations are conflicting.

**Objective:**

The objective of this systematic review and meta-analysis is to assess the effect of the two SNPs on the ADRB2 gene, in terms of the responses that present in asthmatic patients shortly after albuterol inhalation.

**Methods:**

The primary outcome of this work is a detailed study of the associations of the two SNPs in the ADRB2 gene with treatment response and lung function testing shortly after administration of albuterol to asthmatic patients. A comprehensive literature search, using the OVID platform, MEDLINE, EMBASE, and the Cochrane Central Register of Controlled Trials, will be conducted by a specialized librarian without language restrictions. We will include both prospective and retrospective original observational studies, and we will exclude nonhuman or in vitro studies. All abstracts will be reviewed by two authors who will also individually perform data extraction from each eligible study. Any arising disagreements will be resolved through discussion with a third party. Risk of bias for all included studies will be independently assessed using the quality of genetic association studies tool. We will report the systematic review and meta-analysis, following the Preferred Reporting Items for Systematic Reviews and Meta-Analyses statement. A narrative synthesis of study results or meta-analyses will be undertaken when appropriate.

**Results:**

At the moment of writing, we have already started the preliminary literature search and piloting of the study selection process. The anticipated completion date is September 30, 2019.

**Conclusions:**

Our systematic review and meta-analysis aims to clarify the current evidence of associations between the two nonsynonymous SNPs in the ADRB2 gene and the responses that present in asthmatic patients shortly after albuterol inhalation. If positive correlations are found, this knowledge may be used to improve personalized pharmacotherapy of albuterol use.

**Trial Registration:**

PROSPERO CRD42019074554; https://www.crd.york.ac.uk/prospero/display_record.php?RecordID=74554

**International Registered Report Identifier (IRRID):**

PRR1-10.2196/14759

## Introduction

Albuterol is one of the most commonly used medications in current practice. According to the 2017 Quintiles IMS National Prescription Audit, albuterol is ranked 9th in terms of number of times dispensed in the US market, with 70 million prescriptions in 2016 [1]. Albuterol is a β-2 adrenergic agonist and is used for patients who develop bronchospasm due to conditions such as asthma, chronic obstructive lung disease, and cystic fibrosis [2,3].

Albuterol works by activating β-2 adrenergic receptors on airway smooth muscles, leading to the activation of adenylcyclase and to an increase of cyclic-3’, 5’-adenosine monophosphate (cAMP) in the cells [3,4]. These increased cAMP levels then activate protein kinase A, which inhibits the phosphorylation of myosin and lowers the concentrations of ionic calcium in the cells, thus resulting in the relaxation of airway smooth muscles. In addition, the increased levels of cAMP inhibit the release of mediators from mast cells in the airway. Various kinds of adverse drug reactions occur due to albuterol, including headaches, tachycardia and dizziness, and reported frequencies of these events appears to be greater than 3.0% [3]. Albuterol causes cardiac effects by stimulating β-2 adrenoceptors and by activating peripheral receptors, but it also causes dizziness by stimulating skeletal muscle β-2 adrenoceptors [5,6].

Clinically, the response to albuterol is measured by physical examination with auscultation but forced expiratory volume in the first second (FEV1) can also be used in order to determine both response and reversibility of airflow obstruction after inhalation of albuterol. Reversibility can be measured by observing the change in FEV1. Various cutoff levels of positive response to albuterol have been reported, but usually a 12-15% increase is considered to be a meaningful change [7,8].

It is very common for clinicians to experience interindividual variabilities of responses to albuterol that cannot be explained just by age, severity of asthma, or environmental factors. Some studies estimated that 60.6% of interindividual variation in the FEV1 response due to albuterol may be attributed to genetic factors [9-11]. The first pharmacogenomic study of albuterol came out in 1997 [12], and since then, multiple pharmacogenomic research studies related to albuterol response or adverse reactions and β-2 adrenergic receptor polymorphisms have been reported, most of which have focused on the β-2 adrenergic receptor (ADRB2) gene that is located on chromosome 5q31-q32 and encodes β-2 adrenergic receptors [13]. These studies mainly investigated two polymorphic loci in the ADRB2 gene, including nonsynonymous variants such as Arg16Gly (substitution of arginine to glycine at position 16; rs1042713) and Glu27Gln (substitution of glutamic acid to glutamine at position 27; rs1042714), the minor allele frequencies of which are reported to be 48% for Arg16Gly and 20% for Glu27Gln per 1000 genomes among individuals, according to the Phase 3 Genomes Browser [14]. These two variants were originally reported to be associated with the development of other types of asthma, such as nocturnal asthma, and also with agonist-promoted downregulations by isoproterenol in the in vitro studies [15,16]. The in vitro functional study of those mutations showed that the Arg16Gly mutation had a higher degree of agonist-promoted downregulation of β-2 adrenergic receptor expression after 24 hours of exposure of beta-stimulant [16]. Subsequently, Martinez et al first showed the association of positive response for patients carrying Arg-16 due to albuterol, and they also showed a strong linkage disequilibrium between the two polymorphisms (Arg-16 and Gln-27 alleles) [12]. Another in vitro study also reported these associations by using human airway smooth muscle cells, suggesting that the Gly-16 allele is associated with enhanced downregulation of β-2 adrenergic receptors in transformed cell lines [17]. These in vitro studies could explain the mechanisms of these polymorphisms, in particular a possibility of change in function of β-2 adrenergic receptors which has since been corroborated by other studies [18-22]. However, there are also multiple studies that have showed conflicting results about associations between responses due to albuterol and these polymorphisms, with some showing better response in patients with Gly-16 and others showing no associations with either of these two SNPs [23-29].

Despina et al attempted to conduct a systematic review of these associations back in 2006, but finally decided to abandon this work after concluding that the reported studies were too heterogeneous to standardize and synthesize for systematic review [30]. Subsequently, Finkelstein et al conducted a meta-analysis in 2009, showing positive correlations in asthmatic children with the Arg/Arg genotypes at position 16 of the ADRB2 gene (odds ratio [OR] 1.77; 95% CI 1.01-3.1; *P*=.03), compared with the Arg/Gly or Gly/Gly genotypes [31]. They did not find any positive correlations at position 27 of the ADRB2 gene (OR 1.04; 95% CI 0.76-1.42). They successfully completed the meta-analysis, specifically limiting the target populations and phenotypes to asthmatic children and albuterol responders and defining being responsive as an increase of FEV1 by 15%. However, there are multiple limitations in the meta-analysis, most notably that they did not follow the principles of the Cochrane Handbook for Systematic Reviews of Intervention or the Preferred Reporting Items for Systematic Reviews and Meta-Analyses (PRISMA) statement [32,33]. In addition, they did not conduct an assessment of risk of bias, nor did they show the results of testing for heterogeneity using the I^2^ statistic. Third, this study excluded the investigations of the associations in adult populations, and the studies in which being responsive was defined as an increase of FEV1 by 12% to 15.3% [18,26]. Thus, we cannot definitively rule out the possibility that this study is not an accurate summary of the current evidence for these associations. Since then, more pharmacogenomic studies have been performed for these associations; however, no systematic reviews or meta-analyses that included adult populations have yet been published.

Clinical practice should only be revised after careful and thorough critical review of published studies. Systematic reviews and meta-analysis are currently considered to be the highest levels of evidence and therefore would be sufficient to inform a change in current medical practice [34]. Key to the success of this project will be the focus on study data that relates to specific phenotypes due to one drug only, minimizing heterogeneities and maximizing inclusion of previous studies. Our aim will be to clarify the current records of pharmacogenomics studies that focused on albuterol, and whether the ADRB2 genetic polymorphisms have any influence on responses that present in asthmatic patients shortly after albuterol therapy, then to share our findings with clinicians in order to escalate consideration of incorporating these findings into actual medical practice.

## Methods

### Overview

We will perform a systematic review and meta-analysis based on the principles of the Cochrane Handbook for Systematic Reviews of Intervention [32]. The report of the systematic review and meta-analysis will follow the PRISMA statement [33], and current protocol follows the 2015 PRISMA Protocol (see Multimedia Appendix 1) [35].

### Primary Outcome

In this study we will include asthmatic patients of any age group who are also on albuterol therapy, and we will study the responses of ADRB2 variant-type allele carriers to compare these responses to asthmatic patients carrying the ADRB2 wild-type allele. The study outcomes will be associations of Arg16Gly (rs1042713) and Glu27Gln (rs1042714) polymorphisms in the ADRB2 gene with treatment response and lung function testing (FEV1) shortly after administration of albuterol.

### Search Strategy and Sources

A comprehensive literature search using the OVID platform will be performed by a specialized librarian in order to identify the relevant studies from our respective inception date to completion date, without any special date limits, and using the following electronic databases: MEDLINE, EMBASE, and the Cochrane Central Register of Controlled Trials. No language restrictions will be applied. The literature search strategies will be created using medical subject headings and text words related to “albuterol” AND “ADRB2 gene” AND “asthma”. Various synonyms and related terms for all subjects will be used. Abstracts and conference reports will be included, and articles will also be identified from reference lists, and upon identification of relevant studies the reviewers will check for additional relevant cited, and citing, articles. This search strategy is outlined in more detail in Multimedia Appendix 2.

We registered this protocol on the International Prospective Register of Systematic Reviews on January 24, 2019 (registration number: CRD42019074554). When any amendments are required, we will provide the following information: date of each amendment, description of changes, and rationale for changes. We will include those changes in the protocol.

### Eligibility Criteria

We will include both prospective and retrospective original studies that meet the following criteria: (1) They are observational (cohort, cross-sectional or case-control) studies with control groups, or randomized controlled trials, on asthmatic patients on albuterol therapy; (2) Arg16Gly (rs1042713) and Glu27Gln (rs1042714) polymorphisms in the ADRB2 gene were reported; and (3) short-term differences in change of percentage of FEV1 after albuterol inhalation are reported for relevant genotypes.

We will exclude nonhuman or in vitro studies (experimental), case reports, case series, reviews, editorials, newsletters, commentaries, abstracts, conference reports, and original studies that did not report outcomes of interest. We will exclude studies in which no albuterol, or its equivalents, were used, or no Arg16Gly (rs1042713) or Glu27Gln (rs1042714) polymorphisms in the ADRB2 gene were identified. Duplicated studies will also be excluded.

### Data Collection and Analysis

Studies of patients, regardless of their age, sex, ethnicity, body weight, height, smoking status, duration of asthma, severity of asthma, use of concurrent drugs, and co-morbidities, will be included.

We will extract the eligibility information for each study pertaining to study identification (first author, year of publication, and country where patient recruitment took place), study design, patient population (number of enrolled patients, age, gender, predominant ethnicity, concomitant drug use, smoking status, severity of asthma, co-morbidities, subjects’ genotype of ADRB2 gene), brand or generic name of albuterol, dose of albuterol, whether or not methacholine provocation was conducted, timing to measure lung function testing, genotyping methods, allele frequencies, and results of lung function testing for each genotype of ADRB2. Studies or articles retrieved from all databases will be imported into EndNote. Titles and abstracts of all studies retrieved as a result of the search, as well as those from additional sources, will be screened independently by two review authors (KH and SK) to identify studies that potentially meet the inclusion criteria outlined above. After obtaining full-text versions of all potentially eligible studies, two review team members (KH and SK) will independently assess them for eligibility. Both reviewers will be independently involved in all stages of study selection, data extraction, and risk of bias assessment. Any disagreement between reviewers regarding the eligibility of particular studies will be resolved through discussion with a third independent reviewer (TK). Where studies are duplicated, we will use the study that had the largest number of patients. We will contact the original authors of the studies for missing data by e-mail, when required.

Risk of bias for all included studies will be independently assessed by two reviewers (KH and SK) using the quality of genetic association studies (Q-Genie) tool [36]. This tool, containing 11 items, was developed using the Strengthening the Reporting of Genetic Association Studies and Strengthening the Reporting of Genetic Risk Prediction Studies guidelines [37,38]. The Q-Genie tool has a 7-point scale, rating and classifying 1 and 2 as low, 3 and 4 as moderate, and 5–7 as high. The overall quality of the study is classified by the total score from each question, indicating poor quality if scores ≤35, moderate quality if scores >35 and ≤45, and good quality if scores >45. We will also check for departure from Hardy-Weinberg equilibrium using Michael H. Court’s online calculator [39]. Any disagreement between the review authors will be resolved by consulting with a third reviewer (TK). If necessary, we will contact the original authors of the studies for assistance with clarification of any identified discrepancies. Potential publication bias will be analyzed through the use of a funnel plot and the Egger test.

Data will be analyzed by RevMan 5.3 [40], and characteristics of included studies will be described. A narrative synthesis of study results will be undertaken, including evidence tables and forest plots to aid in data presentation when appropriate. When there is missing data, we will attempt to contact the original authors of the study to obtain the relevant missing data. Where appropriate, imputation methods will be used if missing data cannot be obtained [41]. Meta-analysis of results will be performed if sufficient clinical and statistical data is available, and in that case, individual study results will be pooled (with weights based on the inverse variance method) when two or more studies have similar study designs and have usable data for the outcomes of interest. We will then pool all the results using a fixed effect or random effects meta-analysis. The types of summary statistics considered in the meta-analyses will be standardized mean differences for continuous outcomes and pooled risk ratios or odds ratios for binary outcomes, with 95% CI and two-tailed *P* values for each outcome, based on the following genotypes: Arg/Arg, Arg/Gly and Gly/Gly for Arg16Gly, and Glu/Glu, Glu/Gln, and Gln/Gln for Glu27Gln. *P* values<.05 will be considered significant overall associations of the two polymorphisms, with change of percentage of FEV1 shortly after albuterol use.

Measures of heterogeneity of effect for different studies will be evaluated using the I^2^ statistic and Chi-squared test, when possible. We will consider an I^2^ value greater than 50% indicative of substantial heterogeneity, but if study estimates have small or moderate heterogeneity they will be combined using a DerSimonian and Laird random effects model. When systemic narrative synthesis is performed, it will cover the information presented in the text and tables that is sufficient enough to summarize and explain the characteristics and findings of the included studies.

Publication bias will be examined based on visual inspection of a funnel plot, with mean differences plotted on the x-axis and inverse of variance of the effect plotted on the y-axis.

If the necessary data is available, we will carry out subgroup analyses based on age, year of publication, sample sizes, and whether bronchoconstriction was provoked by methacholine or not. We will also conduct sensitivity analyses to assess heterogeneity, according to quality components and risk of bias, by omitting one study at a time and calculating a pooled effect size for the remaining studies.

## Results

Preliminary literature search and piloting of the study selection process has been started and is anticipated to be completed by September 30, 2019.

## Discussion

We will perform a systematic review and meta-analysis and currently do not anticipate any issues with the implementation of the proposed protocol. A key part of our strategy is to exclusively focus on specific phenotypes due to a certain drug, thus decreasing heterogeneities. Our findings will clarify the current status of pharmacogenomics studies of albuterol response and uncover the limitations of current evidence that are preventing clinical implementation of these findings in actual clinical practice. The work may also facilitate further research and continued accumulation of further evidence for these associations, and if positive correlations are found it could potentially help clinicians to provide more personalized medicinal care for their patients.

## Appendix

Multimedia Appendix 1 PRISMA-P 2015 Checklist.

Multimedia Appendix 2 Search terms and strategies.

## Abbreviations

Arg: arginine
cAMP: cyclic-3’, 5’-adenosine monophosphate
FEV1: forced expiratory volume in the first second
Gln: glutamine
Glu: glutamic acid
Gly: glycine
OR: odds ratio
PRISMA: Preferred Reporting Items for Systematic Reviews and Meta-Analyses
Q-Genie: quality of genetic association studies
