# Supplementary material for: The Influence of Beta-2 Adrenergic Receptor Gene Polymorphisms on Albuterol Therapy for Patients With Asthma: Protocol for a Systematic Review and Meta-Analysis
Source: JMIR Res Protoc. 2019 Sep 16;8(9):e14759. doi: 10.2196/14759 (PMC6773362; doi:10.2196/14759)
Supplement: Multimedia Appendix 2 [file resprot_v8i9e14759_app2.docx]

**Multimedia Appendix 2: Search terms and strategies**

1. **Search strategy for MEDLINE**

1 exp Asthma/ (115842)

2 asthma*.tw. (136428)

3 or/1-2 (156459)

4 Receptors, Adrenergic, beta-2/ (3941)

5 (((adrenergic adj5 receptor*) or adrenoceptor* or adrenoreceptor*) and (beta-2 or beta2)).tw. (11132)

6 (ADRB2* or BAR or B2AR* or ADRBR* or beta2AR* or beta2-AR* or beta-2-AR*).tw. (24229)

7 or/4-6 (33464)

8 3 and 7 (1515)

9 exp Polymorphism, Genetic/ (233113)

10 exp Genotype/ (347598)

11 (gene* or geno*).tw. (4335169)

12 (arg16 or arg 16 or 16arg or 16 arg or arg16gly* or gly16arg* or argb16 or arg b16 or b16arg or b16 arg or position 16).tw. (878)

13 (rs1042713 or rs52812686 or rs3729940 or rs17839749 or rs17846639 or rs17334242 or rs3182174 or rs17287432 or rs56964295 or rs17859732 or rs17334179 or rs17721693).tw. (50)

14 or/9-13 (4418035)

15 8 and 14 (636)

16 exp Albuterol/ (9386)

17 albuterol.tw. (2353)

18 salbutamol.tw. (6866)

19 or/16-18 (12369)

20 15 and 19 (153)

21 exp animals/ not humans.sh. (4326005)

22 20 not 21 (149)

1. **Search strategy for EMBASE**

1 'asthma'/exp AND [embase]/lim NOT [medline]/lim (88171)

2 asthma*:ab,ti AND [embase]/lim NOT [medline]/lim (68350)

3 #1 OR #2 (94199)

4 'beta 2 adrenergic receptor'/de AND [embase]/lim NOT [medline]/lim (1749)

5 (adrenergic NEAR/5 receptor*):ab,ti OR adrenoceptor*:ab,ti OR adrenoreceptor*:ab,ti AND β2:ab,ti AND [embase]/lim NOT [medline]/lim (1011)

6 adrb2*:ti,ab OR bar:ti,ab OR b2ar*:ti,ab OR adrbr*:ti,ab OR β2ar*:ti,ab AND 'β2 ar*':ti,ab AND [embase]/lim NOT [medline]/lim (208)

7 #4 OR #5 OR #6 (2543)

8 #3 AND #7 (507)

9 'genetic polymorphism'/exp AND [embase]/lim NOT [medline]/lim (87096)

10 'genotype'/exp AND [embase]/lim NOT [medline]/lim (112851)

11 gene*:ti,ab OR geno*:ti,ab AND [embase]/lim NOT [medline]/lim (1503976)

12 arg16:ti,ab OR 'arg 16':ti,ab OR 16arg:ti,ab OR '16 arg':ti,ab OR arg16gly*:ti,ab OR gly16arg*:ti,ab OR argb16:ti,ab OR 'arg b16':ti,ab OR b16arg:ti,ab OR 'b16 arg':ti,ab OR 'position 16':ti,ab AND [embase]/lim NOT [medline]/lim (295)

13 rs1042713:ti,ab OR rs52812686:ti,ab OR rs3729940:ti,ab OR rs17839749:ti,ab OR rs17846639:ti,ab OR rs17334242:ti,ab OR rs3182174:ti,ab OR rs17287432:ti,ab OR rs56964295:ti,ab OR rs17859732:ti,ab OR rs17334179:ti,ab OR rs17721693:ti,ab AND [embase]/lim NOT [medline]/lim (55)

14 #9 OR #10 OR #11 OR #12 OR #13 (1526536)

15 #8 AND #14 (223)

16 'salbutamol'/de AND [embase]/lim NOT [medline]/lim (10826)

17 salbutamol:ti,ab AND [embase]/lim NOT [medline]/lim (3059)

18 albuterol:ti,ab AND [embase]/lim NOT [medline]/lim (1411)

19 #16 OR #17 OR #18 (11263)

20 #15 AND #19 (62)

21 #20 NOT ([animals]/lim NOT [humans]/lim) (60)

1. **Search strategy for Cochrane Central Register of Controlled Trials**

1 MeSH descriptor: [Asthma] explode all trees (9972)

2 asthma*:ti,ab,kw (26232)

3 #1 or #2 (26232)

4 MeSH descriptor: [Receptors, Adrenergic, beta-2] this term only (119)

5 (((adrenergic adj5 receptor*) or adrenoceptor* or adrenoreceptor*) and (beta-2 or beta2 or β2)):ti,ab,kw (546)

6 (ADRB2* or BAR or B2AR* or ADRBR* or beta2AR* or beta2-AR* or beta-2-AR* or β2AR* or β2-AR*):ti,ab,kw (5140)

7 (rs1042713 or rs52812686 or rs3729940 or rs17839749 or rs17846639 or rs17334242 or rs3182174 or rs17287432 or rs56964295 or rs17859732 or rs17334179 or rs17721693):ti,ab,kw (8)

8 #4 or #5 or #6 or #7 (5168)

9 #3 and #8 (1811)

10 MeSH descriptor: [Polymorphism, Genetic] explode all trees (3536)

11 MeSH descriptor: [Genotype] explode all trees (5010)

12 (gene* or geno*):ti,ab,kw (126244)

13 (arg16 or arg 16 or 16arg or 16 arg or arg16gly* or gly16arg* or argb16 or arg b16 or b16arg or b16 arg or position 16):ti,ab,kw (1315)

14 (rs1042713 or rs52812686 or rs3729940 or rs17839749 or rs17846639 or rs17334242 or rs3182174 or rs17287432 or rs56964295 or rs17859732 or rs17334179 or rs17721693):ti,ab,kw (8)

15 #10 or #11 or #12 or #13 or #14 (127522)

16 #9 and #15 (208)

17 MeSH descriptor: [Albuterol] explode all trees (2762)

18 albuterol:ti,ab,kw (3492)

19 salbutamol:ti,ab,kw (3769)

20 #17 or #18 or #19 (5508)

21 #16 and #20 (82)

22 #21 in Trials (71)
